# The Roles of Epithelial-to-Mesenchymal Transition (EMT) and Mesenchymal-to-Epithelial Transition (MET) in Breast Cancer Bone Metastasis: Potential Targets for Prevention and Treatment

**DOI:** 10.3390/jcm2040264

**Published:** 2013-11-22

**Authors:** Binnaz Demirkan

**Affiliations:** Division of Medical Oncology, Department of Internal Medicine, Faculty of Medicine, Dokuz Eylul University, Izmir 35340, Turkey; E-Mail: binnaz.demirkan@deu.edu.tr; Tel.: +90-232-4124803

**Keywords:** breast cancer bone metastasis, epithelial-to-mesenchymal transition (EMT), mesenchymal-to-epithelial transition (MET), prevention, treatment

## Abstract

Many studies have revealed molecular connections between breast and bone. Genes, important in the control of bone remodeling, such as receptor activator of nuclear kappa (RANK), receptor activator of nuclear kappa ligand (RANKL), vitamin D, bone sialoprotein (BSP), osteopontin (OPN), and calcitonin, are expressed in breast cancer and lactating breast. Epithelial-mesenchymal transition (EMT) and mesenchymal-epithelial transition (MET) effectors play critical roles during embryonic development, postnatal growth, and epithelial homeostasis, but also are involved in a number of pathological conditions, including wound repair, fibrosis, inflammation, as well as cancer progression and bone metastasis. Transforming growth factor β (TGFβ), insulin-like growth factor I & II (IGF I & II), platelet-derived growth factor (PDGF), parathyroid hormone-related protein (PTH(rP)), vascular endothelial growth factor (VEGF), epithelial growth factors II/I (ErbB/EGF), interleukin 6 (IL-6), IL-8, IL-11, IL-1, integrin αvβ3, matrix metalloproteinases (MMPs), catepsin K, hypoxia, notch, Wnt, bone morphogenetic proteins (BMP), and hedgehog signaling pathways are important EMT and MET effectors identified in the bone microenviroment facilitating bone metastasis formation. Recently, Runx2, an essential transcription factor in the regulation of mesenchymal cell differentiation into the osteoblast lineage and proper bone development, is also well-recognized for its expression in breast cancer cells promoting osteolytic bone metastasis. Understanding the precise mechanisms of EMT and MET in the pathogenesis of breast cancer bone metastasis can inform the direction of therapeutic intervention and possibly prevention.

## 1. Introduction

Over 80% of the malignant tumors are of epithelial origin and a number of these carcinomas are highly osteotropic. More than 90% of cancer-related death is caused not by the primary tumor, but by their metastases at distant sites [1,2].

Although breast cancer mortality has been declining over the past decade, primarily due to earlier detection, adjuvant therapies, and the advent of targeted therapies for estrogen receptor-positive and (epithelial growth factor receptor-2) HER2-positive cancers; many patients relapse after an initial response to standard treatment options. Bone is the most common site of breast cancer metastasis (70%) [3,4,5,6,7,8,9]. 

Bone metastasis is associated with reduced survival, impaired quality of life, and pain due to skeletal-related events (SREs) [10]. Well-known bone-targeted agents, bisphosphonates and the receptor activator of nuclear kappa ligand (RANKL) inhibitor, denosumab, have emerged as effective options for the treatment of breast cancer patients with bone metastases [11,12,13,14,15,16,17,18]. 

Tumors contain a subpopulation of cells, called cancer stem cells (CSCs), which have the ability to self-renew and regenerate the tumor. The residual tumors after systemic treatments (hormonotherapy, chemotherapy, or targeted therapy) are enriched for CSCs and have a gene signature with hallmarks of epithelial-mesenchymal transition (EMT)-like properties [19,20,21,22]. Epithelial-mesenchymal transition (EMT) endows metastatic properties upon cancer cells to promote invasion, migration, and subsequent dissemination. The disseminated tumor cells recruited into the target organs may also undergo mesenchymal-epithelial transition (MET) that would favor metastasis formation [23,24,25,26].

This article will review the clues and clinical implications of EMT and MET for treatment and possibly prevention of bone metastasis of breast cancer. 

## 2. The Clues of Epithelial-Mesenchymal Transition

### 2.1. The Role of Peripheral Blood Circulating Tumor Cells and Bone Marrow Disseminated Tumor Cells

Much of the evidence for a possible role of EMT in progression of breast cancer and bone metastasis has arisen from studies of *in vitro* culture of epithelial cell lines. *In vivo* and clinical evidence has started to accumulate recently.

Metastasis is associated with the presence of peripheral blood circulating tumor cells (CTCs) and bone marrow disseminated tumor cells (DTCs) in patients with breast cancer [26,27,28]. Early in the metastatic cascade, cancer cells from the primary tumor acquire invasive properties and gain access to the blood or lymphatic vascular systems, which is aided by neo-angiogenesis and remodeling/destruction of the basement membrane. In the bloodstream (and presumably in lymphatic vessels), intravasated CTCs are capable of surviving and eventually reach “hospitable” distant secondary sites, such as bone, lungs, brain, and liver. Extravasation of CTCs at the secondary site requires recognition of, and adhesion to, vascular endothelial cells followed by matrix degradation [26,29,30,31,32,33,34]. Finally, the CTCs must invade the secondary tissue to become DTCs, typically shown in the bone marrow. All of these processes are evidence of a more motile and plastic “mesenchymal like” phenotype that promotes movement from a syncytial mass and invasion through tissue [35,36].

An EMT-like process, first described in embryonic development, is one of the main mechanisms involved in breast cancer metastasis and most likely contributes to metastases from all types of carcinomas [37].

### 2.2. EMT Classification

EMT can be classified into three subtypes. Type 1 EMT occurs during development and includes the mesenchymal transition of primitive epithelial cells during gastrulation, generation of migrating neural crest cells from neuroepithelial cells, and formation of endocardial cushion tissue from cardiac endothelial cells. Type 2 EMT includes the transition of secondary epithelial (and endothelial) cells to tissue fibroblasts, which can be observed during the processes of wound healing, regeneration, and fibrosis in adult tissues. Type 3 EMT also occurs in adult tissues and involves the mesenchymal transition of epithelial carcinoma cells, leading to generation of metastatic tumor cells [38].

### 2.3. Breast Cancer Stem Cells (BCSCs) and Mesenchymal Stem Cells (MSCs)

BCSCs were originally described by Al-Hajj *et al*., in 2003 [39]. They isolated a tumorigenic subset of cancer cells from human breast tumors based on the expression of the surface markers CD44+, CD24−/low, and ESA+ (CD, cluster of differentiation; ESA, epithelial specific antigen). 

The CD44 high/CD24 low phenotype in breast cancer cell has been linked to EMT through the mesenchymal attributes of breast cancer stem cells, which also have dramatically enhanced malignant properties [40,41,42,43,44].

Stephen Paget, in 1889, proposed the seed and soil hypothesis: Bone provides the fertile soil in which certain cancer cell seeds prefer to grow [45].

Bone is a dynamic tissue that is constantly remodeled through the resorption of old bone by osteoclasts and the subsequent formation of new bone by osteoblasts [46,47].

MSCs from BM (bone marrow) can become tumor-associated fibroblasts, have immunosuppressive function, and facilitate metastasis by epithelial-to-mesenchymal transition. Moreover, MSCs generate osteoblasts and osteocytes and regulate osteoclastogenesis. Therefore, MSCs can play an important pro-tumorigenic role in the formation of a microenvironment that promotes BM and bone metastases. MSCs are multipotent progenitor cells, which do not only regulate haematopoietic development, but also give rise to a majority of BM stromal cell lineages. These lineages include osteoblasts, adipocytes, chondrocytes, fibroblasts, endothelial cells, and myocytes. MSCs also release soluble factors that regulate the development and function of osteocytic and osteoclastic lineages, such as IL-1b, IL-6, IL-11, Dkk-1 (Dickkopf-1), Wnt proteins (Wnt 2, 4, 5, 11, 16), TGF-β, FGF-2, PDGF, PGE2, RANKL, LIF, OPG, M-CSF, MIP-1a, and HA. As MSCs may have an essential role in invasion and proliferation of cancer cells, there is close interaction and crosstalk among metastatic cancer cells, the BM microenvironment, and bone [48,49,50,51,52,53]. Bone-derived TGF-β is one of the most abundant growth factors in bone matrix and is a major factor regulator of tumor cell behavior in bone [54,55].

### 2.4. EMT Effectors and Signaling Pathways

TGF-β itself is a regulator of both physiological and pathophysiological EMT. TGF-β elicits its cellular responses by binding to TGF-β type I and type II serine/threonine kinase receptors and phosphorylation of receptor regulated (R-) Smad2 and Smad3. Activated R-Smads form heteromeric complexes with common mediator Smad4, which accumulates in the nucleus, where they control gene expression in a cell type-specific manner [56,57,58,59,60,61,62].

Normal mammary gland development is under the influence of hormones, such as estrogen, progesterone, and prolactin, during the stages of prepuberty, puberty, pregnancy, lactation, and involution. A number of genetic pathways control this process, including the RANK/RANKL/OPG pathway. Studies have also demonstrated a key role for the RANK/RANKL/OPG pathway during mammary tumor formation and metastasis [63,64,65,66,67,68]. 

RANKL is a TNF family member that activates NF-κB and plays a fundamental role in antigen-presenting cells and during osteoclastogenesis. RANKL protein is exclusively expressed in PR-positive cells in the mammary epithelium. RANKL is necessary for the extensive proliferation of HR-negative epithelial cells in response to progesterone stimulation [68]. Another paracrine mediator of PR signaling is calcitonin, a 32-amino-acid peptide hormone involved in calcium homeostasis. Calcitonin expression is induced by progesterone in the luminal cells. The cognate calcitonin receptor is expressed in the myoepithelium, suggesting that calcitonin may act as a paracrine factor in a heterotypic interaction; but its biological function remains to be defined [69]. Taken together, ER and PR, they function in the mammary involvement of the stroma in the case of pubertal ER signaling and possibly more crosstalk with myoepithelial cells in PR-driven epithelial proliferation [63,66].

RANKL has been proposed to act as a paracrine mediator of stem cell activation because expression of its cognate receptor, RANK, is enriched in the basal compartment. 

Type I, type II, and type III or atypical cadherins are expressed in the mammary gland. Type I cadherins include epithelial (E), neural (N), placental (P), and retinal (R) cadherins. E-Cadherin is expressed exclusively in all of the mammary epithelial cells, while P cadherin is expressed in mammary epithelial cells of the alveoli and ducts, but also in the myoepithelial cells. N-Cadherin is expressed in mesenchymal cells of the mammary stroma. R-Cadherin, which was first identified in the retina, is expressed in the mammary epithelial cells. E-Cadherin provides a tight connection between epithelial cells and localizes and interacts with components of the adherens junction [70,71].

The functional loss or downregulation of E-cadherin (CDH1) from epithelial cells is considered a hallmark of EMT. E-Cadherin downregulation in cancer cells often occurs as a result of promoter methylation. The dissolution of adherens junctions is a critical step of EMT, with loss/decrease or relocalization of CDH1 as the most commonly used determinant of the EMT phenotype [72,73,74,75,76].

All metastatic tumors of invasive ductal carcinoma were seen to be re-expressing E-cadherin irrespective of the E-cadherin status of the primary tumors [71]. Studies, thus, provide proof of principle that the metastatic cascade invokes E-cadherin emergence and, thus, supports a MET-like phenomenon.

Vimentin is a key regulator of breast cancer cell migration and a marker for mesenchymal subtype, characteristic of cancer cells that have undergone epithelial-mesenchymal transition. Expression of vimentin is related to reduced expression of E-cadherin and upregulation of N-cadherin [77].

MicroRNAs can regulate TGF-β-induced apoptotic and growth suppressive functionality. miRNAs together with other non-coding RNAs (long non-coding RNAs, small nucleolar RNAs, and ultraconserved regions) contribute to carcinogenesis. miRNAs can function both as oncogenes and as tumor suppressors, the involvement of different miRNAs is reported in the formation and regulation of human BCSCs. The microRNA (miR)-200 family is master regulator and inhibits initialing steps of EMT and metastasis [78,79,80,81,82,83]. Down-regulation of the epithelial miR-200 family in primary breast tumors, which leads to repression of E-cadherin, already predispose the cancer to successful metastasis, as evidenced in poorer outcomes [79]. The miR-200 family members have been revealed to promote E-cadherin re-expression via the repression of ZEB family genes, causing inhibition of cancer invasion and metastasis [80,81]. Expression of miR-200 is shown to be epigenetically regulated by histone-modifications and DNA promoter methylation. While let-7 family, miR-200 family, miR-30 family, miR-128, miR-34c, and miR-16 are downregulated in BCSCs; miR-181 and miR-495 are upregulated in BCSCs. The most frequently cited EMT-related miRNAs are those belonging to the miR-200 family, which consists of miR-200a/b/c, miR-141, and miR-429. miR-10b is also highly expressed in metastatic breast cancer cells and promotes tumor invasion and migration [84,85,86,87].

Locally invading tumor cells undergoing an EMT proliferate less as they migrate more. EMT can arrest cell proliferation through many EMT regulators such as β-catenin, Snail, and ZEBs [88,89,90].

Bone marrow-derived human MSCs promote *de novo* production of lysyl oxidase (LOX) from human breast carcinoma cells, which is sufficient to enhance the metastasis of otherwise weakly metastatic cancer cells to the lungs and bones.

LOX is a copper-dependent amine oxidase that catalyzes the cross-linking of collagens and elastins in the ECM. LOX is an essential component of the CD44-Twist signaling axis, in which extracellular hyaluronan causes nuclear translocation of CD44 in the cancer cells, thus, triggering LOX transcription by associating with its promoter. Processed and enzymatically active LOX, in turn, stimulates Twist transcription, which mediates the MSC-triggered epithelial-to-mesenchymal transition (EMT) of carcinoma cells. Surprisingly, although induction of EMT in breast cancer cells has been tightly associated with the generation of cancer stem cells, it is shown that LOX, despite being critical for EMT, does not contribute to the ability of MSCs to promote the formation of cancer stem cells in the carcinoma cell populations [91].

Release of secreted proteins (termed the secretome) appears to underline the progression of the metastatic phenotype [92]. For example, secretion of soluble cytokines and chemokines is known to modulate cell-cell communication at primary and secondary tumor sites, however, a novel suite of extracellular vesicles (EVs) (exosomes) capable of horizontal transfer of information (protein, mRNA, miRNA, and lipid) between cells has been identified as important regulators of the tumor microenvironment [36,93]. 

EVs have been implicated in modifying the tumor microenvironment to induce angiogenesis and metastasis in breast cancer, as well as facilitate the transfer of oncogenic potential through activation of MAPK and Akt signaling pathways. Given that EVs carry disease specific signatures such as miRNAs, their clinical importances are being investigated.

Tumor-associated macrophages regulate breast cancer invasiveness through exosome-mediated delivery of oncogenic miRNAs (oncomiRs). Exosomes commonly contain expression of proteins involved in multivesicular body (MVB) biogenesis (TSG101, Alix), heat shock proteins (Hsp70, Hsp90), cytoskeletal components (actins, tubulins, keratins), adhesion molecules (integrins, tetraspananins), and membrane trafficking regulators (Rabs, annexins). Exosomes can modulate the immune response, control stromal remodeling in the metastatic niche, activate signaling pathways in neighboring cells, and transfer genetic and oncogenic information to recipient cells, increase cell motility [93,94,95,96,97].

Vimentin and TGF-β regulates MTHFD2 (methylenetetrahydrofolate dehydrogenase 2) expression in metastatic breast cancer cells. It has been shown that MTHFD2 knockdown reduces cancer stem cell properties of bone metastatic breast cancer cells. Mitochondrial enzyme MTHFD2 has a potential role in breast cancer progression and bone metastasis [77,98]. 

Overexpression of mesenchymal genes, such as SPARC (secreted protein acidic and rich in cysteine), indicates that breast cancer cells may acquire mesenchymal markers by EMT and by fusion with MSCs; in particular, SPARC has recently been associated with the most aggressive and highly metastatic tumors [36,99].

The serine/threonine kinase protein kinase D1 (PKD1) in normal ductal epithelial cells of the breast maintains the epithelial phenotype and prevents epithelial-to-mesenchymal transition (EMT). In addition to its inhibitory effects on EMT, PKD1 negatively affects directed cell migration by blocking actin reorganization processes at the leading edge of migrating cells. Furthermore, the expression and activity of PKD1 regulate the invasiveness of breast cancer cell lines by inhibiting the expression of multiple matrix metalloproteinases (MMPs) [100]. 

Except for CDH1 (E-cadherin), a variety of proteins that are down-regulated in response to an EMT include plakoglobin (JUP), occludin (OCLN), zonula occludens1 (TJP1), α-catenin (CTNNA3), and claudins 3/4/7 (CLDN-3/4/7). On the other end of the spectrum, the promotion of a mesenchymal-like phenotype is indicated by the up-regulation of proteins such as fibronectin (FN1), CDH2, VIM, ACTA2, and nuclear CTNNB1. The zincfinger proteins Snail1 (SNAI1), Snail2 (SNAI2), Zeb1 (ZEB1), and Zeb2 (ZEB2) each directly repress transcription of CDH1 in mammary cells by binding the E-boxes (CANNTG) located in the CDH1 proximal promoter, as do the basic helix-loop-helix factors E12/E47 (TCF3) and TWIST1. A number of other transcription factors cause relocalization of junctional CDH1, including SIX1, goosecoid (GSC), and forkhead box C2 (FOXC2). Interestingly, knockdown of CDH1 alone is sufficient to induce an EMT, highlighting the significance of repressors of CDH1 in the induction of an EMT. Indirect repression of CDH1 is also accomplished by EMT inducers, including SIX1, GSC, and FOXC2. Recently, p53 (TP53), Twist2 (TWIST2), and forkhead box Q1 (FOXQ1) have been added to this list of oncogenic EMT inducers [22,100]. 

The transcription factor Runx2 is essential for the formation of the skeleton and it also has a role in the regulation of normal mammary gland gene expression such as the transcription of the mammary gland-specific gene, β-casein. This skeletal transcription factor is aberrantly expressed at high levels in breast cancer cells that aggressively metastasize to the bone environment. In cancer cells, Runx2 activates expression of bone matrix and adhesion proteins, matrix metalloproteinases, and angiogenic factors that have been associated with metastasis [101,102,103,104]. In addition, Runx2 mediates the responses of cells to signaling pathways hyperactive in tumors, including BMP/TGFβ and forms co-regulatory complexes with SMADs and other co-activator and co-repressor proteins to regulate gene transcription contributing to tumor growth in bone and the accompanying osteolytic disease [46]. 

**Figure 1:**
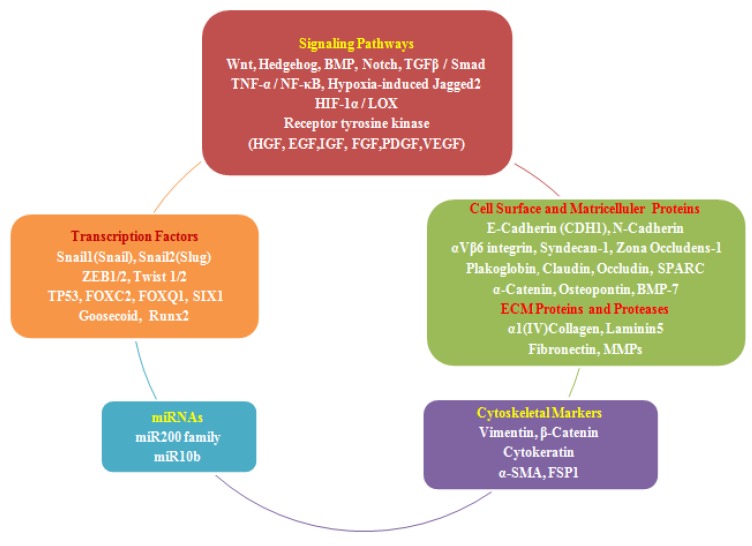
Signaling pathways and markers of epithelial to mesenchymal transition (EMT).

In addition to transcription factors, several signaling pathways are known to induce an EMT, such as the TGF-β/Smad, receptor tyrosine kinases (epithelial growth factor (EGF), hepatocyte growth factor (HGF), insulin-like growth factor (IGF), fibroblast growth factor (FGF), platelet-derived growth factor (PDGF), vascular endothelial growth factor (VEGF)), bone morphogenetic proteins (BMP), Wnt, notch and hedgehog, TNF-α/NF-κB, hypoxia-induced Jagged2, and HFI-1α/LOX pathways [105,106,107], Figure 1.

## 3. The Clues of Mesenchymal-Epithelial Transition

A process opposite to the initial EMT at the primary tumor site, mesenchymal to epithelial transition (MET), is an evolving and relatively under-investigated mechanism that is considered to contribute substantially to the colonisation of DTCs into metastatic tumors at the secondary site. Recent studies suggest that MET can occur in breast cancer models. As E-cadherin (CDH1) downregulation in cancer cells often occurs as a result of promoter methylation, loss of promoter methylation at the secondary site causes the metastatic cancer cells to re-express E-cadherin through MET [108,109,110,111,112,113,114,115,116,117,118]. A potential demethylating factor has been identified as 1α,25-dihydroxyvitamin D3, which has been shown to promote *de novo* E-cadherin re-expression in breast cancer cell lines [119,120]. Microenvironmental factors like miRNAs can also contribute to MET at the metastatic site. It is shown that miR-200 promotes Sec23A-positive secretory vesicles, the cargo of which may regulate both autocrine and paracrine pathways to promote establishment, survival, and/or growth of the macrometastases [121,122,123,124,125,126,127,128].

**Figure 2:**
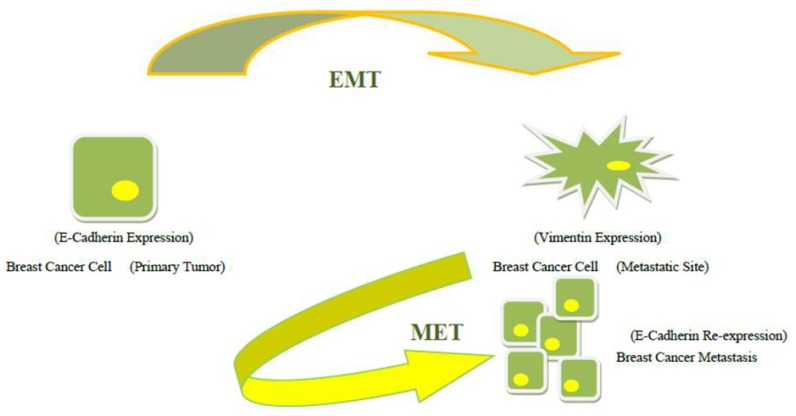
Mesenchymal to epithelial transition (MET).

EMT and MET may determine dormant or active states of the tumor, respectively, and allow for an indeterminate number of cycles of invasion and metastases formation, Figure 2.

## 4. Clinical Implications of EMT and MET-Driven Growth of Metastases

Evidence suggests that EMT-associated apoptosis reduction and senescence inhibition contribute largely to therapeutic resistance. Cells that have undergone EMT withstand external insults better, leading these cells to display resistance to chemotherapy and endocrine therapy [19,20,21,22,117,129,130,131]. 

Tumors that have undergone a MET at a secondary site may be more susceptible to apoptotic insults and, hence, may be treated more successfully with chemotherapeutic drugs. Subclinical tumor may be forced to undergo a MET to facilitate therapy.

Thus, it is reported that experimental model systems will be needed to settle this key question as it directly impinges on whether inducing or inhibiting MET would be beneficial in the treatment of breast cancer. Further, the question of whether the MET is stable in the metastases or if these cells show ongoing phenotypic plasticity leading to a second EMT is also open to question.

Elimination of tumor cells that exhibit a mesenchymal phenotype could potentially be achieved by blocking the signaling pathways that trigger and/or maintain tumor EMT. In particular, blockade of the IL-8–IL-8R axis appears to be an attractive strategy to disrupt the autocrine positive feedback loop between EMT and IL-8, while simultaneously decreasing the paracrine signals that mesenchymal tumor cells could exert on their surrounding environment. Secretion of IL-8 is also a feature of the tumor stroma, and blockade of IL-8 signaling could be fundamental in lessening the tumor promoting signals originated on stromal fibroblasts, monocytes, neutrophils, and endothelial cells in response to stressful environments, including hypoxia, acidosis, or genotoxic damage. Supporting this strategy, several preclinical studies have already demonstrated the ability of neutralizing antibodies to the IL-8Rs, a humanized antibody against IL-8 (ABXIL-8) and the small-molecule inhibitor repertaxin to inhibit angiogenesis, tumor growth, and metastasis in xenograft tumor models [132]. 

Members of TGF-β superfamily, which include bone morphogenetic proteins (BMP), are involved in the control of many different biological processes, including cell proliferation, differentiation, apoptosis, and regulation of invasiveness. The homodimeric protein BMP7 induces MET in normal and nontransformed cells.

BMP7 expression in patients with primary breast tumors exclusively developing bone metastases is significantly lower than in primary breast tumors developing exclusively visceral (lung and/or liver) metastases. These clinical findings suggest that decreased BMP7 expression may confer a bone metastatic potential to human breast cancer cells. Normal ducts of the breast display strong apical BMP7 protein expression. Functional studies reveal that BMP7 overexpression by breast cancer cells inhibits *de novo* formation of osteolytic bone metastases and, hence, the metastatic capability of breast cancer cells in *in vivo* bone metastasis model. BMP7 is able to counteract SMAD-dependent TGF-β signaling. Inactive TGF-β is concentrated and stored in high amounts in extracellular bone matrix and can be released and activated by osteoclastic resorption. Activated bone matrix-derived TGF-β may act as a paracrine growth factor for neighboring osteolytic cancer cells that may have colonized the bone marrow. BMP7 regulates epithelial homeostasis in the human mammary gland by preserving the epithelial phenotype. Decreased BMP7 expression during breast cancer progression may, therefore, contribute to the acquisition of a bone metastatic phenotype. Furthermore, exogenous BMP7 can still inhibit breast cancer growth at the primary site and in bone marrow. Therefore, BMP7 may represent a novel therapeutic molecule for repression of local and bone metastatic growth of human breast cancer [133,134].

Related with the bone resorption induced by breast tumor cells, there is evidence that the TRAF inhibitor ABD56 is able to inhibit the M-CSF and RANKL induced osteoclastogenesis enhanced by breast tumor cells. ABD56 acts both on osteoclast precursor blocking the membrane localization and ubiquitination of TRAF6 and subsequent phosphorylation of various factors induced by RANKL as IkB, and on breast tumor cells inhibiting the adhesion, spreading, and migration with no impact on the cell viability. Such dual effect of TRAF inhibitor, not observed for RANKL inhibitor and different from the previously described for bisphosphonates, suggests great therapeutic potential for such inhibitors. There is also evidence that the VEGF inhibitor sunitinib can normalize vascularization of highly osteolytic bone metastatic tumor and improve efficacy of the associated cytotoxic therapy. Jagged1, which is expressed by osteoblasts under the control of by parathyroid hormone signaling pathway co-opted in pathological bone metastasis, can regulate the expansion of hematopoietic stem cells in the bone microenvironment through Notch signaling. The clinical importance of Jagged1 is its association with an increased incidence of breast cancer relapse and bone metastasis. It is shown to be an ideal target for monoclonal antibody therapy as it is a cell-surface protein. TGF-β signaling has been targeted by therapeutic agents currently being tested in clinical trials [135,136,137,138,139,140,141,142,143].

EMT transcription and signaling pathways are considered as anticancer drug targets. For EMT transcription pathways; AKT (KTX-O401 (perifosine), VQD-OO2 (API-2), GSK690693, mTOR (RAD 001 (everolimus)), XL-765 (Exelixis)), NF-κβ (OT-304, IMD-0354), β-catenin (ERX-3722), PKC (LY317615 (enzastaurin)), and for EMT signaling pathways; EGFR-1 (erlorinib, gefitinib), ErbB2 (trastuzumab), IGF-1R (CP-751, 871; AMG479), VEGF/VEGFR (bevacizumab, cediranib) Src (dasatinib, bosutinib), and NOTCH (anti-notch-1 monoclonal antibody) are investigated [100,143].

## 5. Limitations

Search stategy is restricted with the EMT/MET pathways and breast cancer bone metastasis and treatment or prevention. Only published English-language articles of preclinical and clinical studies as well as reviews were considered eligible.

## 6. Conclusions

There is growing implication of EMT and MET in the progression of breast carcinoma and bone metastases both in studies with experimental models and humans. New surrogate markers are needed to define different stages during the transition from the epithelial to mesenchymal phenotype, and the reverse transition.

The interrelationship between CSCs, embryonic signaling pathways, and EMT/MET pathways offers a continuum of potential therapeutic targets for breast carcinoma related bone diseases. Theraupetic delivery of microRNAs, “differentiation-inducing” agents such as HDAC inhibitors, antibody-directected to the cytoskeletal markers or manipulating the skeletal transcription factors, such as Runx2, by depletion either chemically or by RNA interference can be potential strategies to treat and/or prevent breast cancer bone metastases.
